# Integrated transcriptomic analysis of *Trichosporon Asahii* uncovers the core genes and pathways of fluconazole resistance

**DOI:** 10.1038/s41598-017-18072-9

**Published:** 2017-12-19

**Authors:** Haitao Li, Congmin Wang, Yong Chen, Shaoqiang Zhang, Rongya Yang

**Affiliations:** 1 Department of Dermatology, PLA Army General Hospital, 5 Nanmencang, Beijing, 100700 China; 2 Department of Biological Sciences, Center for Systems Biology, The University of Texas at Dallas, Richardson, TX 75080 USA; 3 College of Computer and Information Engineering, Tianjin Normal University, Tianjin, 300387 China

## Abstract

*Trichosporon asahii* (*T*. *asahii*) has emerged as a dangerous pathogen that causes rare but life-threatening infections. Its resistance to certain antifungal agents makes it difficult to treat, especially for patients undergoing long-term antibiotic therapy. In this study, we performed a series of fluconazole (FLC) perturbation experiments for two *T*. *asahii* strains, a clinical isolate stain CBS 2479 (T2) and an environmental isolate strain CBS 8904 (T8), to uncover potential genes and pathways involved in FLC resistance. We achieved 10 transcriptomes of T2 and T8 that were based on dose and time series of FLC perturbations. Systematic comparisons of the transcriptomes revealed 32 T2 genes and 25 T8 genes that are highly sensitive to different FLC perturbations. In both T2 and T8 strains with the phenotype of FLC resistance, the processes of oxidation-reduction and transmembrane transport were detected to be significantly changed. The antifungal susceptibility testing of FLC and penicillin revealed their resistance pathways are merged. Accumulated mutations were found in 564 T2 and 225 T8 genes, including four highly mutated genes that are functionally related to the target of rapamycin complex (TOR). Our study provides abundant data towards genome-wide understanding of the molecular basis of FLC resistance in *T*. *asahii*.

## Introduction

Since the first identification and usage of the antibiotic penicillin to control microbial infections, resistance to a broad range of antibiotic drugs has become a widespread and severe problem that has achieved great concern and global attention^1–3^. Although bacterial and eukaryotic microorganisms share similar biological responses to drug exposure, fungal pathogens pose a particularly interesting challenge as fungi are more similar to their hosts than prokaryotic pathogens in terms of their biochemistry, metabolism and genetic composition^4,5^. Worldwide, fungi infect billions of people and kill more than 1.5 million per year, exhibiting a staggering impact on human health^6^. *T*. *asahii* is a rare but emerging fungal pathogen that causes severe invasive infections, especially in immunocompromised patients undergoing long-term antibiotic treatment typically lasting weeks or months^7^. *T*. *asahii* has achieved drug resistance to most antifungals such as amphotericin B, flucytosine, caspofungin and floconazole (FLC)^8–10^, which seriously limits clinical options and results in high mortality rates. However, only one gene of *T*. *asahii*, the lanosterol 14α-demethylase, has been identified in azole resistance^11^. It is urgent and important to systematically investigate the core genes, pathways and mechanisms of drug resistance in *T*. *asahii*.

Several cellular mechanisms have been associated with drug resistance in fungi based on the studies of *Saccharomyces cerevisiae* (*S*. *cerevisiae*)^12^, *Candida albicans* (*C*. *albicans*)^13,14^ and *Aspergillus fumigatus (A*. *fumigatus)* ^15^, including the increasing efflux of a drug from a cell^16^, the cellular alterations that minimize the toxicity of the drug and the alteration or amplification of the drug target^17,18^. For example, mutation or over-expression of Erg11 has been reported in azole resistance in *C*. *albicans* ^18^ and *A*. *fumigatus* ^15^. In echinocandins resistance in fungi, mutation of the target of Fks1 is widely detected^17^. It is obvious that these mechanisms are not working separately but in a combinational way. The combinability of different resistance mechanisms is the main factor determining the maximum level of resistance possible and how soon drug resistance arises^4,5^. *T*. *asahii* has been reported to achieve stable resistance to several antifugal agents in only weeks^8,9^. Connecting the evolution of drug resistance with the compounded mechanisms could ultimately reveal how pathogens adapt to new antibiotics and would be beneficial for precisely treating the infections of *T*. *asahii* through combination therapy.

In order to understand the dynamic responses of FLC perturbations and accumulated mutations of protein coding regions in FLC-resistant strains, we performed the first systematic transcriptome analysis of FLC resistance by using time series and comparative analysis of two *T*. *asahii* strains. We discovered genes and pathways whose expressions were dramatically changed in FLC-resistant cells. We also detected tens of alternative splicing (AS) events and a large number of accumulated mutations at protein-coding regions and 5′ or 3′ untranslated regions (5′ UTR or 3′ UTR), of which four super mutated genes were related to TOR signalling. Furthermore, our antifungal susceptibility testing of FLC and penicillin revealed their resistance pathways are merged. This data delineated a systematic pattern of core genes and pathways that could be involved in FLC resistance in *T*. *asahii* and thus provides a data source towards further studies.

## Results

### The dynamic landscape of gene expressions in clinical isolate strain T2

**Figure 1:**
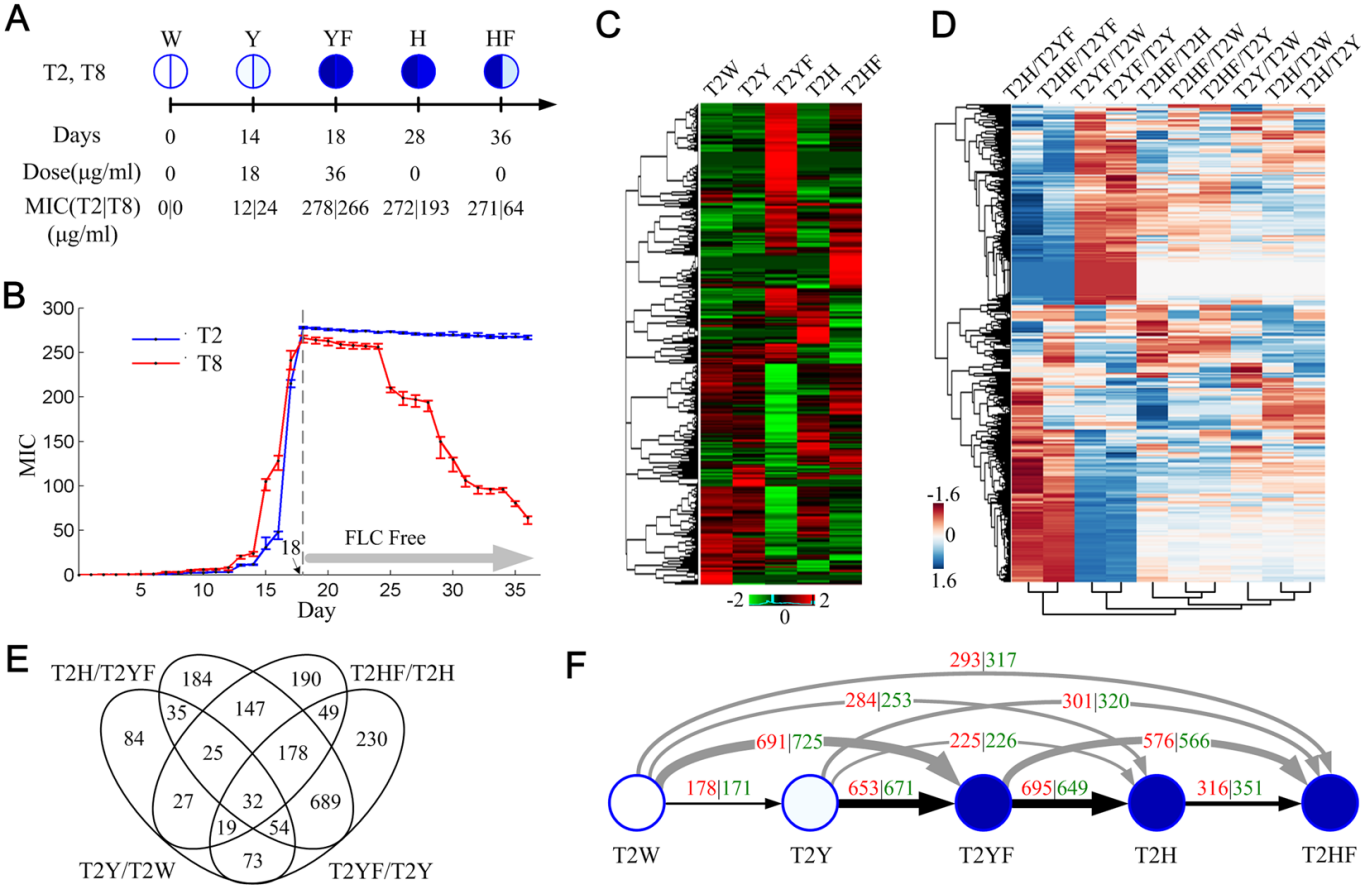
Comparative analysis of T2 transcriptomes. (**A**) Information of sequenced samples of T2 and T8. The darker colours show higher MIC values. W: wild type. Y: induced. YF: force induced. H: rescued. HF: force rescued. (**B**) The relationship of MIC and cultured days for T2 and T8. After 18 days, samples were cultured in FLC free PDA medium. (**C**) The histogram shows the landscapes of T2 gene expressions under five conditions. The z-scores of gene FPKMs are shown. A total of 2281 genes that were of FPKM > = 0.5 for at least one condition were clustered into blocks whose genes have similar expression patterns. (**D**) The histogram shows the landscapes of 2281 gene dynamics (FC) for any of the compared pairs of five conditions. The FCs of the 2281 genes and 10 condition comparisons were hierarchically clustered using a biclustering approach. (**E**) The Venn diagram shows the comparisons of four DEG sets (|log_2_FC| > 1). (**F**) Systematic comparisons of the DEG sets of any compared condition pairs. The red numbers indicate the up-regulated expressions (log_2_FC > 1), and green numbers indicate the down-regulated expressions (log_2_FC < −1). The line thickness indicates the total number of up-regulated and down-regulated genes.

We performed a dose and time series of FLC perturbation experiments and observed different resistance levels in T2 (CBS 2479) and T8 (CBS 8904) cells. T2 is a standard strain of *T*. *asahii* that was isolated in 1929 from a patient with progressive psoriasis but had not been exposed to FLC, and its genome was sequenced in 2012^19^. T8 is an environmental strain that was isolated from maize cobs in 1998 and was also sequenced in 2012^20^. When the cells were cultured with increasing doses of FLC, the Minimum Inhibitory Concentration (MIC) of T2 and T8 were observed to be increasing slowly (Fig. 1A,B). After 14 days, the MICs of T2 and T8 started to increase dramatically. When the cells were cultured for 18 days, the MICs of T2 and T8 had increased to >278 and >266, indicating that the cells have the clear phenotype of FLC resistance (Supplementary Fig. S1A). We then did negative selections on these cells by culturing them in medium without FLC. Surprisingly, we found that the MICs of T8 cells decreased over the time of culturing, while the MICs of T2 kept unchanged (Fig. 1B and Supplementary Fig. S1B). These experiments clearly confirmed that the T2 and T8 cells could rapidly achieve drug resistance during FLC induced experiments. Interestingly the T2 cells kept the stable ability of FLC resistance and T8 cells are partially reversible.

To identify the genes involved in FLC resistance, we used the RNA-Seq method to detect the gene expressions of five condition points that have significant phenotypes (Please see Fig. 1A,B, Supplementary Fig. S1A,B and Supplementary Table S1 for detailed explanations of the five conditions and phenotypes). First, we successfully achieved the high quality of transcriptomes with high coverage depth and high mapping quality; 96.35~96.81% of the raw reads were mapped to the genome (Supplementary Table S1). To better compare the gene expression values among different samples, we calculated the Fragments Per Kilobase of transcript per Million mapped reads (FPKM) and normalized the FPKM by using the z-score method^21^ (Supplementary Fig. S2A). We confirmed that the genome-wide gene expressions of different samples have high correlations, indicating the RNA-Seq experiments are coordinate and successful (Pearson correlations > 0.86, Supplementary Fig. S2B). After filtering the genes that were not expressed in any of the 5 conditions (FPKM < 0.5), a total of 2281 genes were observed in at least one condition. The clustering of these genes exhibited clear blocks whose genes have similar expressions, indicating dramatic and coordinated switches of cellular functions (Fig. 1C). Next, we calculated the fold changes of the genes by comparing across the 5 conditions to understand their expression dynamics. By using a hierarchical clustering method^22^ for both genes and compared condition pairs, we achieved not only the gene sets that have similar dynamic patterns but also the condition pairs with similar or reversed gene expression landscapes (Fig. 1D). Interestingly, we found the genes dynamic patterns of two pairs, T2H/T2YF vs T2HF/T2YF and T2YF/T2 vs T2YF/T2Y, have high similarities (Pearson Correlation 0.96 and 0.97), however dynamic patterns between four pairs are reversed (Pearson Correlations as −0.86, −0.85, −0.89, −0.83 for T2H/T2YF vs T2YF/T2W, T2H/T2YF vs T2YF/T2Y, T2HF/T2YF vs T2YF/T2W and T2HF/T2YF vs T2YF/T2Y respectively), indicating reversed regulations of cellular functions. Considering our sequential experiments are based on different transactions of FLC perturbations, we especially compared the gene sets whose expressions are highly changed in a sequential way (T2W- > T2Y- > T2YF- > T2H- > T2HF, |log_2_FC| > 1) (Fig. 1E,F). In total, 688 genes were observed to be differentially expressed in at least one condition transaction (Supplementary Table S2) and 32 genes were common among these comparisons, suggesting they are very sensitive to FLC dose dynamics. We then systematically compared the transcriptomes for any of the condition pairs. This type of comparison is also important since we can find the differentially expressed genes (DEG) based on different backgrounds of FLC treatments. Overall, we found a large number of genes with different expressions for 10 comparisons (|log_2_FC| > 1, Fig. 1F). We observed that four comparisons related to T2YF have high numbers of DEG, among which the change from T2W to T2YF achieved the largest number: 1416 (see Supplementary Table S3 for a summary and Supplementary Table S2 for detailed information of gene fold changes). These results indicate that, under the T2YF condition, the cells exhibited dramatic changes of genome-wide expression patterns and were consistent with our MIC observations that T2 cells achieved the highest MIC value after culturing in FLC medium for 18 days.

We then performed detailed functional analysis and pathway analysis for the four gene sets that have differential expressions during four sequential transactions. Considering there is no available database of T2 and T8 for direct functional analysis, we aligned the genes of T2 and T8 onto the genome of *S*. *cerevisiae* (Yeast) and used their Yeast homologs for functional analysis. In total, 2721 genes of 8835 T2 genes have homologs in the Yeast genome, while the union of the four gene sets includes 688 genes mapping to 553 Yeast homologs (Supplementary Table S4). We found that the genes are significantly enriched in the biological processes of transmembrane transport, oxidation-reduction process and metabolic process during the four sequential transactions (P-value < 1e-3, see Supplementary Table S5 for a complete list). This result was consistent with the observation from the enrichment analysis of the KEGG pathways that these genes are highly enriched in metabolic pathways, biosynthesis of antibiotics and fatty acid degradation (P-value < 1e-2). The analysis of cellular components shows that these genes are significantly related to the plasma membrane and the integral component of plasma membrane (P-value < 1e-3). Meanwhile, the molecular functions are mainly enriched in oxidoreductase activity and transporter activity (P-value < 1e-3). These results suggest that, in FLC medium, the *T*. *asahii* cells would speed up multiple metabolic processes and transmembrane transport to reduce the FLC density in the cytoplasm, which is consistent with previous studies from the Yeast system^16^.

### The dynamic landscape of gene expressions in environmental isolate strain T8

Since T2 is a clinical isolated strain, its expression patterns could have been perturbed by the clinical treatments. To obtain a more clear background of FLC resistance, we also collected the transcriptomes of T8, which is an environmental isolated strain. As with the analysis of T2, we first confirmed the high quality of transcriptomes with high coverage depth and high mapping quality; 95.33~96.78% of the raw reads were mapped to the genome (Supplementary Table S1). We normalized the gene FPKMs among different samples and observed high correlations of gene expressions among different samples (Pearson correlations > 0.86, Supplementary Fig. S2A,B). There are a total of 1704 genes observed to be expressed in at least one condition after filtering the genes that are not expressed in any of the 5 conditions (FPKM < 0.5). The clustering of these genes exhibited clear blocks of genes that have similar expressions (Supplementary Fig. S3A). We then calculated the fold changes of the genes by comparing across the 5 conditions. When clustering these dynamic patterns of different condition pairs, we observed the condition pairs which have similar or reversed gene expression landscapes (Supplementary Fig. 3B). We compared the gene sets whose expressions are highly changed (|log_2_FC| > 1) in a sequential way (T8W- > T8Y- > T8YF- > T8H- > T8HF) and detected 481 genes that have differential expressions in at least one condition transaction, including 25 genes with differential expressions for four condition transactions (Supplementary Fig. S3C and Supplementary Table S2). We further compared all the combinatorial pairs of transcriptomes and discovered a large number of genes with different expressions based on different condition background (|log_2_FC| > 1, Supplementary Fig. S3D and Supplementary Table S2). We then performed detailed GO-term analysis and pathway analysis for the 481 genes by using their yeast homologs (Supplementary Table S4) and achieved very similar results as T2 (Supplementary Table S5). For example, these genes are significantly enriched in the biological processes of transmembrane transport, oxidation-reduction process and metabolic process (P-value < 1e-3). However, no significant biological processes or pathways were detected during the transaction from T8W to T8Y.

### The core genes and pathways of FLC resistance in *T*. *asahii*

**Figure 2:**
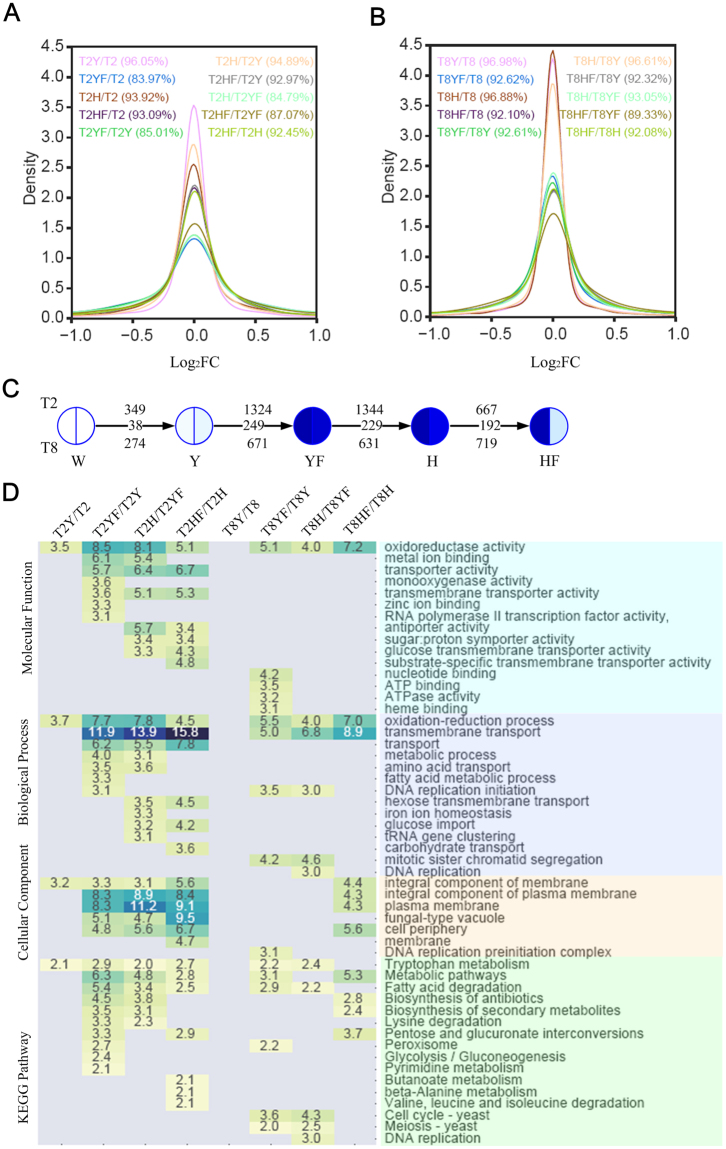
The comparison of T2 and T8 transcriptomes. (**A**) Log_2_FC density distributions of 10 T2 comparing pairs. The numbers show the percent of genes with |log_2_FC| < 1 among all genes. (**B**) Log_2_FC density distributions of 10 T8 comparing pairs. (**C**) The sequential comparisons of five conditions of T2 and T8. The numbers above the arrow line are the numbers of DEG genes of T2; the numbers under the arrow line are the numbers of DEG genes of T8; the numbers on the arrow line are the number of common DEG genes of T2 and T8. The darker blues show the higher MIC values. (**D**) Histogram of significantly enriched biological processes, molecular functions, cellular components and KEGG pathways. The numbers show the −log_10_(P-value). The gray colour indicates no significance detected.

We next compared the gene expressions and dynamics of T2 and T8 to obtain the core genes and pathways of FLC resistance since these comparisons would further filter the noise of strain-specific genes. First, we found that the genes of T2 exhibit more dynamic responses to FLC perturbations than T8 genes. We calculated the proportion of DEG genes among the total genes of T2 and T8 for ten comparisons. The DEG genes of T2 were tested to be significantly larger than those of T8 (Supplementary Table S2. P-value < 0.05, Wilcoxon Signed-Ranks Test, one-tailed). Meanwhile, the proportions of T2 genes that have no differential expressions (|log_2_FC| < 1) were clearly observed to be less than those of T8 (Fig. 2A,B). Second, we compared the DEG genes of T2 and T8 by considering only their homologs. In total, we found there are 38, 249, 229 and 192 homologs of T2 and T8 for the four condition transactions respectively (Fig. 2C, Supplementary Table S6), which dramatically reduced/filtered the DEG numbers of T2 and T8. For example, there are 1324 and 671 DEG genes for T2 and T8 under the condition transaction Y- > YF, but 249 homologs between them. Considering that both T2YF and T8YF achieved the phenotype of FLC resistance, these 249 genes would include high potential candidates involved in the drug resistance of *T*. *asahii*. Furthermore, three T2 genes (A1Q1_07978, A1Q1_08092 and A1Q1_05738) and their T8 homologs were detected within all four DEG sets (Table 1). Since their expressions are highly sensitive to FLC perturbations, they could be core players in FLC resistance. For example, A1Q1_07978 was annotated as a carboxypeptidase, the protein family which has been widely used for antibiotic targets such as Penicillin-binding proteins (PBPs)^23^. Here A1Q1_07978 has very high sequence similarity with a penicillin-binding protein KLP13355 in *Fusarium fujikuroi* (identity 96.9% and E-value 1e-152). Considering previous studies had observed several carboxypeptidases to be involved in beta-lactam resistance^24–26^, vancomycin resistance^27^ and resistance against nitric oxide^28^, our results suggest that A1Q1_07978 could be involved in FLC resistance in *T*. *asahii*.

**Table 1:** Three T2 (T8) genes are sensitive to FLC perturbations.

T2	T8	Identity	E-value	Annotation	Yeast Homology
A1Q1_07978	A1Q2_07884	98.43	0	Carboxypeptidase	YJL172W
A1Q1_08092	A1Q2_03759	97.51	1.00E-160	Major Antigen	—
A1Q1_05738	A1Q2_05898	99.07	0	Hypothetical Protein	—

To obtain the potential mechanisms underlying FLC resistance in *T*. *asahii* cells, we systematically compared the enriched biological processes and pathways of T2 and T8. We observed that the biological processes of oxidation-reduction and transmembrane transport were commonly and significantly changed in T2 and T8 cells during the condition transactions. Eight biological processes such as metabolic processes, fatty acid degradation and amino acid transport were only detected to be significantly changed in T2 but not in T8 (Fig. 2D). These results are consistent with the enrichment analysis of molecular functions, where the oxidoreductase activity was common in T2 and T8, but transporter activity, metal ion binding and monoxygenase activity were only enriched in T2 (P-value < 1.0e-3). Meanwhile nucleotide binding, ATP binding, heme binding and ATPase activity were only detected in T8 but not in T2. The pathway analysis from the KEGG pathway database^29,30^ shows that 16 pathways were significantly changed in at least one condition transaction. Among these pathways, 13 pathways are observed to be unique or more significant in T2, including several metabolism pathways such as lysine degradation, butanoate metabolism, beta-alanine metabolism and pyrimidine metabolism. Together, these results show that although T2 and T8 share several common functional categories in achieving FLC resistance, T2 cells exhibit more significant dynamics on diverse biological processes.

### The accumulated mutations in FLC resistant cells of *T*. *asahii*

**Figure 3:**
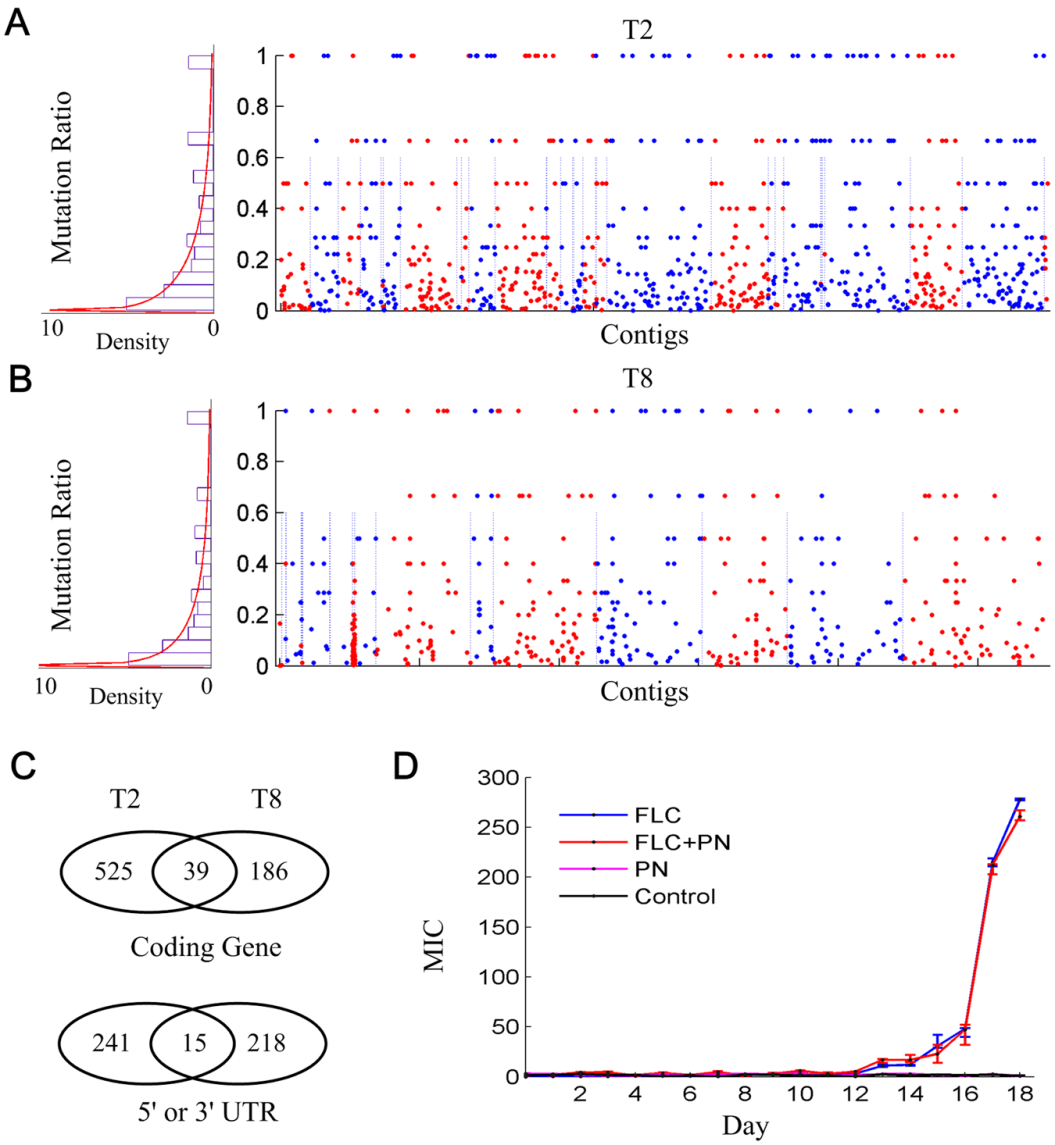
The accumulated mutations in T2YF and T8YF FLC-resistant genomes. (**A**) The relative position and mutation ratio distribution of accumulated mutations in the T2YF genome. The red and blue dots show the alternative contigs. The red line shows the fitted Weibull distribution (shape parameter = 0.2342 with Std. Err. 0.0097 and scale parameter = 0.8533 with Std. Err. 0.0225). (**B**) The relative position and mutation ratio distribution of accumulated mutations in the T8YF genome. The red line shows the fitted Weibull distribution (shape parameter = 0.2136 with Std. Err. 0.0113 and scale parameter = 0.7927 with Std. Err. 0.0247). (**C**) The Venn diagram of mutated genes and 5′ or 3′ UTRs in T2YF and T8YF. (**D**) MIC results of susceptibility testing of FLC and penicillin (PN) on T2 cells. The cells that were cultured in PDA medium without any drug added were termed as control.

One of the major mechanisms of drug resistance in eukaryotic microorganisms is accumulating mutations that alter drug-binding specificity of targeted proteins or affect the pathways/processes by regulating the activities of their protein members^5,31–33^. The drug-resistance mutations have been reported in proteins of 14 α-demethylase enzyme Erg11p^34^, ATP-binding cassette (ABC) transporter Mrpa^35^ and Yor1^36^, and as well as β-1,3 glucan synthase genes Fks1 and Fks2^37,38^. An Erg11p homology in *T*. *asahii* had been reported where a point mutation (G1357C) resulted in a single amino acid substitution at G453R, causing high resistance to azoles treatment^11^. To further investigate landscape mutations related to FLC resistance in *T*. *asahii*, we systematically analyzed mutations from our transcriptomic data by comparing the coding regions of wild type cells and FLC-resistant cells. There are a total of 889 mutations detected in the T2YF genome (Fig. 3A, Supplementary Table S7), including 648 mutations in 564 coding regions (genes) and 241 in 5′ UTRs or 3′ UTRs. For the 648 T2YF mutations in coding regions, 384 mutations were detected as non-synonymous and 165 as synonymous mutations, achieving a non-synonymous/synonymous ratio of 2.33 (384/165). Meanwhile, there are 627 mutations in the T8YF genome (Fig. 3B, Supplementary Table S8), including 409 mutations in 225 coding regions and 218 in 5′ UTRs or 3′ UTRs. For the 409 T8YF mutations in coding regions, 179 mutations were detected as non-synonymous and 230 as synonymous mutations, achieving a non-synonymous/synonymous ratio of ~0.78 (179/230). Surprisingly, we found the non-synonymous/synonymous ratio of the T2YF genome is almost 3 fold of the T8YF genome, suggesting the T2YF genome received higher positive selection of FLC stress than T8YF^39,40^. The mutation ratios of T2YF and T8YF were well fitted as Weibull distribution respectively (Fig. 3A,B left subfigures) which were used for detecting beneficial mutations in cell populations^41,42^. In the T2YF genome, we found 64 mutations with mutation ratio as high as 1 (P-value < 0.05) that are located in 28 genes. In T8YF genome, 53 mutations were found with mutation ratio as high as 1 (P-value < 0.05), which are located in 14 genes.

We then compared the mutated genes of T2YF and T8YF to detect the gene set that were mutated in both strains. Such comparisons are important since the commonly mutated genes could be involved in similar mechanisms of FLC resistance that were independently achieved in T2YF and T8YF. In total, 39 genes were detected, including 9 enzymes, 8 proteins related to transcriptional regulation and 22 hypothetical proteins (Fig. 3C, Supplementary Table S9). Among these genes, 5 genes (A1Q1_01278, A1Q1_02047, A1Q1_03322, A1Q1_03867 and A1Q1_04147) were detected to be with high mutation ratios (>0.25) in both strains and the last four genes were functionally related to the TOR complex/pathway. First, A1Q1_02047 is annotated as 1-phosphatidylinositol-3-phosphate 5-kinase whose mutations were reported to reduced TOR1 activity^43^. A1Q1_03322 is annotated as a hypothetical protein. We aligned its sequence in the CDD database^44^ and the Pfam database^45^, and found it includes a WD-40 domain (cl25539 and pfam00400, E-value 1.83e-07) that is a functional domain in a specific subunit Las24p/Kog1p of TOR1^46^. A1Q1_03867 is a leucine zipper-like protein that includes a Dopey-N domain (cl04407 and Pfam04118, E-value 7.19e-57), which is required for TOR-dependent transcriptional regulation^47,48^. Its homolog Yap1 in *Cryptococcus neoformans* was reported to be required for normal FLC and oxidative stress resistance^49^. A1Q1_04147 is annotated as a hypothetical protein that includes the domain of DNA_pol3_gamma3 super family (pfam12169 and cl26386, E-value 8.46e-10), indicating it could be a PolIII protein. It is also linked to the TOR pathway since ongoing TOR1 signalling is required for PolIII transcription in yeast cells^50,51^. Combined together, we observed that four highly mutated genes are functionally related to the TOR signalling pathway in both T2 and T8 cells, suggesting that inhibition of TOR activity was promoted by FLC stress and, as a feedback, to help accumulating FLC resistance.

### The alternative splicing and lanosterol 14-alpha-demethylase of T2 and T8

It would be interesting to detect the alternative splicing (AS) events that could be involved in FLC resistance, since AS had been observed in multiple fungi of different taxa^52^. We systematically detected the possible alternative splicing events for all comparisons of T2 and T8 cells respectively by using the MATS2.1.0 software^53^. In total, we found 84 AS events by comparing all 5 conditions for T2 cells, including 55 events of mutually exclusive exons (MXE) and 29 events of skipped exon (SE). These AS events were located in 16 genes (Supplementary Table S10). Meanwhile, there are 43 MXE and 6 SE detected in T8 cells, which are located in 13 genes. Comparatively, we found that T2 has more AS events (especially the SE) than that of T8, and the MXE event is the major AS type of T2 and T8. We believe these genes that have AS events occurring could be interesting candidates for further research.

We then checked the T2 and T8 homologs of lanosterol 14 alpha-demethylase, Erg11p in *S*. *cerevisiae* YJM1463, which has been reported to be involved in FLC resistance^34^, to see if they had different expressions, mutations, or AS events under the FLC perturbations. By protein sequence alignments, we detected that its T2 homolog is A1Q1_02098 (Identity 46%, E-value 3.0e-161), and its T8 homolog is A1Q2_04922 (Identity 40%, E-value 2.0e-123). First, we checked their expressions and changes under different conditions. The FPKMs of A1Q1_02098 and A1Q2_04922 show that the two genes are highly expressed and exhibit very similar patterns (Supplementary Fig. S4). However, no high differential expressions of them were observed since the log_2_FC of any paired condition comparisons ranged between −0.5 and 0.5. Second, we checked the mutations of A1Q1_02098 and A1Q2_04922. There are no mutations detected in A1Q1_02098, but a nonsynonymous mutation (C > G) was detected in A1Q2_04922 exon2. This mutation was further observed in the T8H and T8HF genome and thus could be a potential cause by which T8H and T8HF cells achieved phenotype of FLC resistance. Third, we checked the AS events of A1Q1_02098 and A1Q2_04922, but no AS events were found. Finally, we corrected the annotations of A1Q1_02098 and A1Q2_04922 by using our transcriptomic data. A1Q1_02098 was annotated as a protein of 560 amino acids and 5 exons in the NCBI database. However, we found there are 34 amino acids “SRSRVCCFCRSASGSPMPRSTPARTFRRNPTNAQ” with no transcription signal detected in exon3 (Supplementary Fig. S5). This observation is consistent with the translation of its homologous gene Erg11p in *S*. *cerevisiae* YJM1463, which also does not have these 34 amino acids. The reads mapped to the gene A1Q1_02098 clearly shows a decay of the start point at exon3 and follows the GU-AG RNA splicing rule^54^. This evidence highly suggests that the protein sequence of A1Q1_2098 should not have these 34 amino acids included. A similar result was also obtained for the A1Q2_04922 gene.

### The resistance pathways of FLC and penicillin are merged

Our comparative analysis of transcriptomes had revealed that the expressions of penicillin-binding like protein A1Q1_07978 was significantly changed in both T2 and T8 FLC-resistant strains. We hypothesized that penicillin treatment would affect the susceptibility to FLC by affecting A1Q1_07978 activity. To test this, we performed antifungal susceptibility testing of FLC alone, penicillin alone and their combinations against T2 cells. Surprisingly, we found the combination therapy of FLC and penicillin achieved similar MIC results to the FLC monotherapy (Fig. 3D). The FICI score was calculated to be bigger than 2, suggesting there is no synergism of their treatments. This result clearly indicates that the penicillin resistance pathways could have been merged in as one group of FLC resistance pathways. Furthermore, we discovered the penicillin monotherapy had no effects on T2 cells and MIC scores are similar to the controls that were cultured with no drug added (Fig. 3D), indicating that T2 cell could have achieved intrinsic resistance to penicillin.

## Discussion

Systematic understanding of the mechanisms of drug resistance in fungi is an ongoing and challenging problem. It needs a comprehensive and hierarchical investigation of the core genes, pathways and compounded mechanisms of how these contributors interact combinatorially and evolutionarily. As one step to this aim, we obtained and systematically compared the transcriptomes of T2 and T8 strains based on dose and time series experiments of FLC perturbations. The comparative analysis of the transcriptomes and genomes revealed a large number of genes and the common pathways of FLC resistance in T2 and T8 under different conditions. Especially, we identified three novel genes A1Q1_07978, A1Q1_08092 and A1Q1_05738 that are very sensitive to FLC perturbations. Thus, our results described a dynamic landscape of cellular responses during the evolution of FLC resistance in *T*. *asahii* cells.

A large number of clinical studies have proved that certain combination therapies can improve the efficiency of treating fungal infections^55,56^. Our discovery of the core genes and pathways in FLC resistance could offer novel transmission blocking strategies. We found 32 and 25 genes with differential expressions for four condition transactions in T2 and T8 respectively, indicating these genes are very sensitive to FLC perturbations. The functional analysis of these genes shows that they are involved in divergent biological processes/pathways. Thus, the high sensitivity to FLC perturbations and orthogonal functions of these genes indicate they could be good candidates for combinational therapy of *T*. *asahii* infection.

The transcriptomes of T2 and T8 could also be used to complete their draft genomes and improve the annotation of genes. The T2 and T8 strains have only draft genomes finished and are lacking experimental based annotations of genes^19,20^. Our high coverage RNA-Seq data provided the first omics data that could be beneficial for achieving the complete genome and facilitate the annotations of genes, especially the discoveries of novel isoforms. As an example, we have shown the correction of the A1Q1_02098 annotation, and this type of analysis can be performed genome-wide for corrections or confirmations of the *T*. *asahii* genome annotation. The transcriptomes can also be used for detecting AS of genes. We detected 84 AS events for T2 cells and 49 AS events for T8 cells. It is the first time that AS events were reported in the *T*. *asahii* genome. These genes with AS events could be interesting candidates for further research of FLC resistance.

The mutation landscape was also achieved for both T2 and T8 FLC-resistant cells, revealing the potential and gradual effects of FLC stress on the protein activities and transcriptional regulations. We detected 564 and 409 mutations in coding regions of T2 and T8 FLC-resistant genomes respectively, including 39 common genes. Meanwhile there are 241 and 218 mutations found in the 5′ UTRs or 3′ UTRs in the T2 and T8 FLC-resistant genomes respectively, that could affect transcription regulation. Strikingly, four genes with high mutation ratios are functionally related to the TOR pathway, indicating the TOR pathway was evolutionarily modified under FLC stress. Thus, targeting of TOR signalling could be a therapeutic strategy for treating *T*. *asahii* infectious disease that evades resistance^57^. To further study the mutation effects and genes functions, we can manipulate the *T*. *asahii* genome by using homologous recombination with short stretches of sequence homology to delete and mutate genes and to create fusions to epitope tags and fluorescent proteins. For a summary, our results provide abundant data towards genome-wide understanding of the molecular basis of FLC resistance in *T*. *asahii* and may provide clinical options to improve the treating efficiency and reduce the resistance.

## Materials and Methods

### Prepare and culture the cells

Two *T*. *asahii* strains CBS 2479 (T2) and CBS 8904 (T8) with high sensitivity to FLC were selected from the laboratory database of the Department of Dermatology, General Hospital of Beijing Military Region. To systematically investigate the potential genes that could be involved in drug resistance, we performed a conditional and time series of FLC induced experiments that were used to achieve not only FLC resistant cells but also 10 transcriptomes of T2 and T8, where T2 is a clinical isolated strain and T8 is an environmental isolated strain. First, both strains were respectively and serially subcultured in potato dextrose agar (PDA) medium with increasing concentrations of FLC for 18 days (see Supplementary Table S1 for detailed FLC doses). Each sample of different FLC concentrations were cultured under 37 °C for two days and reached a density of approximately 10^8^ cells mL^−1^. Then, the culture medium containing ~10^6^ cells was transferred into fresh PDA medium containing a certain concentration of FLC and re-cultured. For each passage, the E-test method was used to evaluate the susceptibility of cells to FLC, which was indicated by the MIC^58^. The testing of each stage was independently repeated three times. The cells with MIC > 256 μg/ml are considered as FLC induced cells. Second, cells with induced resistance were serially subcultured in drug free PDA medium for 18 days, and a drug susceptibility assay was performed after each subculture.

### RNA-Seq and data analysis

A total of 10 samples were selected for RNA-Seq to obtain the genome-wide gene expression patterns (see Supplementary Table 1 for information of selected samples). We termed the samples T2W, T2Y, T2YF, T2H and T2HF for T2 cells (T8W, T8Y, T8YF, T8H and T8HF for T8 cells) through the paper. RNA was extracted from cell lines using Trizol reagent (Invitrogen), according to the manufacturer’s protocol. The Illumina platform was used for analyzing transcriptomes by employing a 100-bp paired-end library according to the manufacturer instructions (Illumina). The high quality of mRNA samples was confirmed using Agilent 2100 Bioanalyzer total RNA Chip (Agilent) prior to sequencing. Each sample was paired-end sequenced with the Illumina HiSeq. 2000 using HiSeq Sequencing kits. The RNA-Seq data of this study has been submitted to the NCBI GEO database under accession number GSE106454.

FastQC^59^ was used to assess the read quality. We trimmed the low quality reads by using the AfterQC method^60^. Instead of using the default parameters of AfterQC for trimming, we trimmed the left and right end of a read no more than 5 bp respectively to keep the number of reads and read length (>=25) for the following mapping. HiSAT2 software^61^ was used to map the raw reads to T2 and T8 genomes respectively. Stringtie^62^ was used for calculating the FPKM values. Pearson correlations between samples were calculated. The FPKMs were quantile normalized and log2 transformed (termed as Z-score of FPKM)^21^ for the rest of analyses. The log2 fold-change was calculated to compare the expressions of a gene *g* between two samples ${\mathrm{log}}_{2}FC(g)={\mathrm{log}}_{2}\frac{FPKM({g}^{2})+0.5}{FPKM({g}^{1})+0.5}$, where a pseudo count of 0.5 was added to avoid division of zero and to filter low expression values^63^. Genes with |log_2_FC| > 1 were considered as DEGs. Hierarchical clustering^22^ was subjected to gene expressions and the Log_2_FC values to obtain the gene/condition clusters that have similar dynamic patterns.

### Mutation calling and analysis

For the T2YF sample and T8YF sample, we called the mutations after mapping reads against the T2 and T8 genomes. The mutation ratios were calculated as the percentage of the mutation bases at each position using Samtools^64^. The mutation ratios of all mutated positions were fitted as Weibull distribution by using the MATLAB toolbox “dfittool”. The mutations were annotated as nonsynonymous mutation and synonymous mutation by using the SnpEff program^65^.

### Comparative and Functional analysis

The genes with differential expressions were used for the following comparative analysis and pathway analysis. First, the proteins of T2 and T8 were aligned with each other by using the Blastp method^66^. They were then aligned with the proteins of *S*. *cerevisiae* to find the homologous genes. Only the genes with an E-value less than 1e-5 are considered homologs. Second, the genes with differential expressions between two conditions (|log_2_FC| > 1) were subjected to GO enrichment analysis and pathway analysis by considering their yeast-homologs in the DAVID database^67^ and BiNGO^68^. The protein domains were predicted by using conserved domain database (CDD)^44^ and the Pfam protein family database^45^.

### Antifungal susceptibility testing of FLC and penicillin

The activities of FLC and penicillin against T2 cells were evaluated by using a series of their combinations. The T2 cells were serially subcultured in PDA medium with serial concentrations of FLC as describe before (Supplementary Table S1), while keeping a constant concentration of penicillin (4 μg/mL). The T2 cells that were cultured in penicillin or no drug added PDA medium were both used as controls. All tests were performed in triplicate and the T2 activity was expressed as the mean of inhibitions. Wells containing the lowest fractional inhibitory concentrations were used to determine the fractional inhibitory concentration indices (FICI)^69^. FICI = MIC(A2)/MIC(A1) + MIC(B2)/MIC(B1), where A1 is the MIC value of FLC monotherapy, A2 is the MIC value of FLC combination therapy, B1 is the MIC value of penicillin monotherapy, and B2 is the MIC value of penicillin combination therapy). Synergistic effects were defined in interpretations of FICI values as ≤ 0.5 (perfect synergy), >0.5 to <1 (partial synergy), a value of 1 (additive effect), and >1 (loss of synergism).

### Disclosures and Ethics

The authors have read and confirmed their agreement with the authorship of *Scientific Reports* and conflict of interest criteria. The authors declare that they have no conflicts of interest, privacy and confidentiality. The authors have confirmed that this article is unique and not under consideration or published in any other publication, and no copyrighted material are used in this work.

## Electronic supplementary material

Supplementary File 1

Supplementary Table S1

Supplementary Table S2

Supplementary Table S3

Supplementary Table S4

Supplementary Table S5

Supplementary Table S6

Supplementary Table S7

Supplementary Table S8

Supplementary Table S9

Supplementary Table S10
