# Supplementary material for: Integrated transcriptomic analysis of Trichosporon Asahii uncovers the core genes and pathways of fluconazole resistance
Source: Sci Rep. 2017 Dec 19;7:17847. doi: 10.1038/s41598-017-18072-9 (PMC5736589; doi:10.1038/s41598-017-18072-9)
Supplement: Supplementary file 1 [file 41598_2017_18072_MOESM1_ESM.pdf]

Integrated transcriptomic analysis of *Trichosporon Asahii* uncovers the core genes and pathways of fluconazole resistance

Haitao Li[1]*, Congmin Wang[1]*, Yong Chen[2]*, Shaoqiang Zhang[3], Rongya Yang[1§]

[1]Department of Dermatology, PLA Army General Hospital, 5 Nanmencang, Beijing 100700, China

[2]Department of Biological Sciences, Center for Systems Biology, The University of Texas at Dallas, Richardson, TX 75080, USA

[3]College of Computer and Information Engineering, Tianjin Normal University, Tianjin 300387, China

*These authors contributed equally to this work

[§]Corresponding author

Rongya Yang

E-mail: rongyayang@gmail.com

Tel.: 86-10-84008067

Fax: 86-10-84008103

Supplementary File1 includes 5 Supplementary Figures S1-5

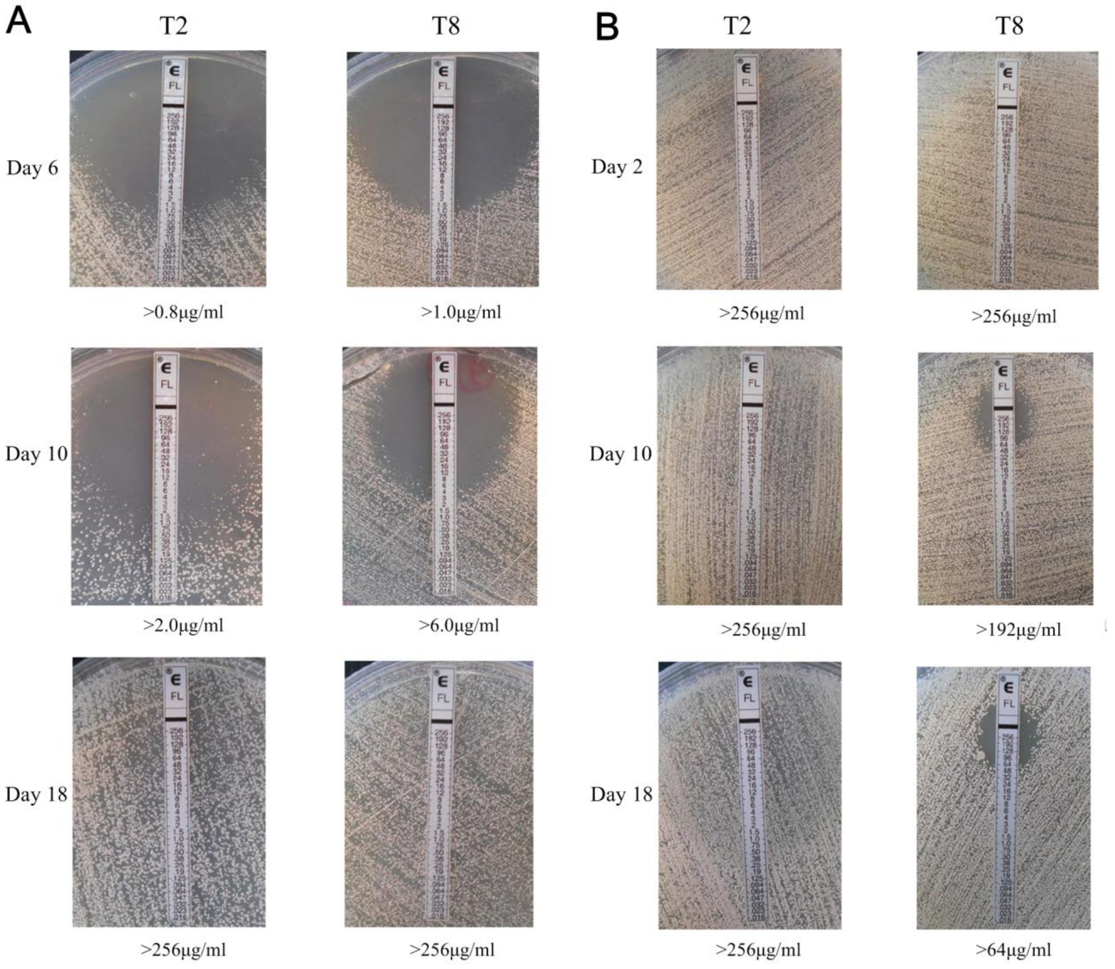

Supplementary Figure S1. FLC resistances under dose and time experiments of T2 and T8. (**A**) The phenotypes of FLC induced experiments for T2 and T8. The MIC scores were tested by using E-test method evaluate the susceptibility of cells to FLC treatment. (B) The phenotypes of rescued experiments for T2 and T8.

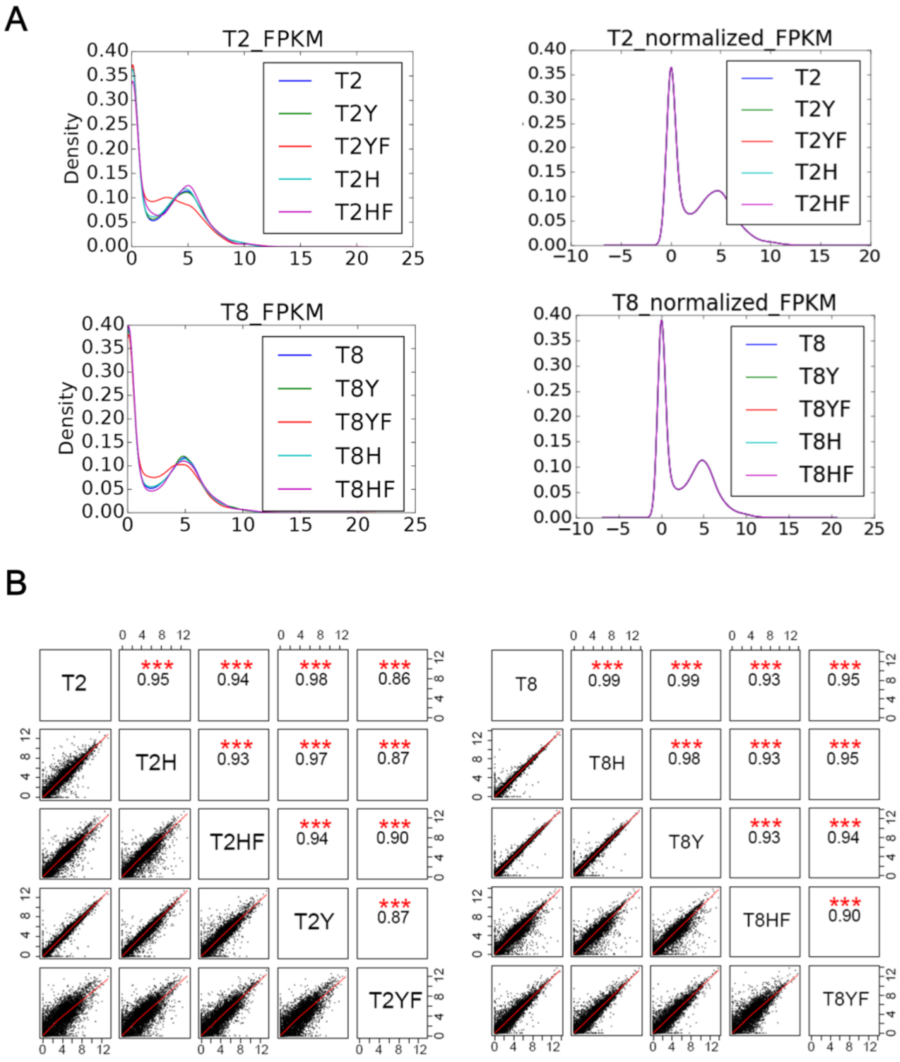

Supplementary Figure S2. Normalization and correlation of gene expressions. (**A**) The raw FPKMs and normalized FPKMs of T2 and T8 genes. (**B**) The correlations of $\log_2$(FPKM+1) of different RNA-Seq results. The numbers show the Pearson correlation.

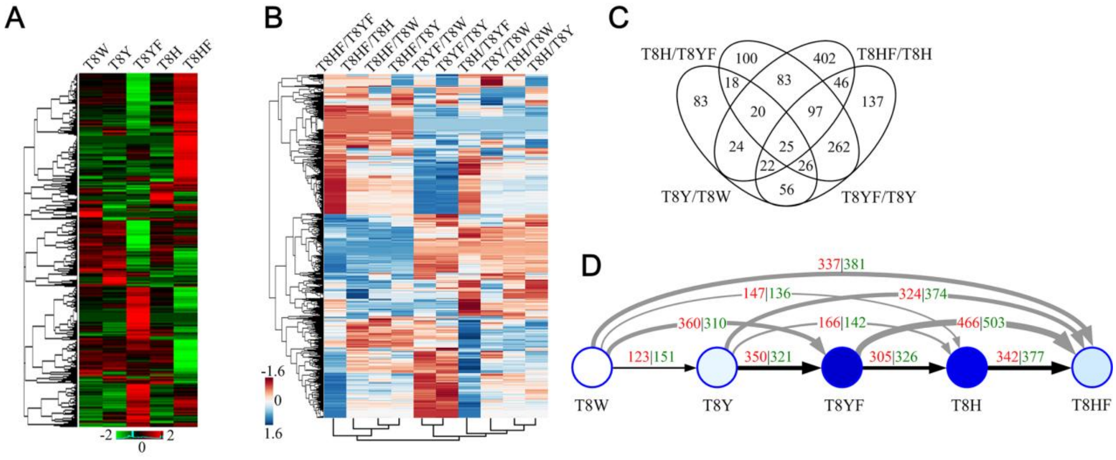

Supplementary Figure S3. Comparative analysis of T8 transcriptomes. (**A**) The histogram shows the landscapes of T8 gene expressions under five conditions. The z-scores of gene FPKMs were shown. A total of 1704 genes that were of FPKM>=0.5 at least one condition were clustered into blocks whose genes have similar expression patterns. (**B**) The histogram shows the landscapes of gene dynamics for any compared pair of five conditions. The FCs of 1704 genes and 10 condition comparisons were hierarchically clustered by a biclustering approach. (**C**) The Venn diagram shows the comparisons of four DEG gene sets ($|\log_2 FC|>1$). (**D**) Systematic comparisons of the DEG sets of any compared condition pairs. The red numbers indicate the up-regulated expressions ($\log_2 FC>1$), and green numbers indicate the down-regulated expressions ($\log_2 FC<-1$). The line thickness indicates the total number of up-regulated and down-regulated genes.

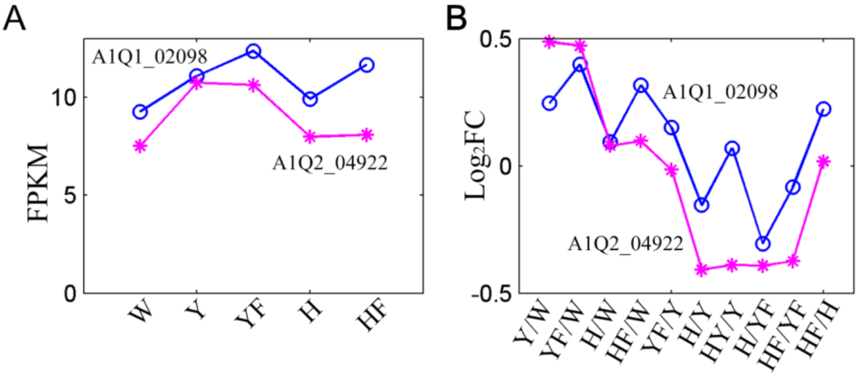

Supplementary Figure S4. The expressions of A1Q1_02098 and A1Q2_04922. (**A**) The FPKMs of five conditions. W: wild type. Y: induced. YF: force induced. H: rescued. HF: force rescued. (**B**) The sequential comparisons of five conditions. Later time-points were compared with all its previous time-points.

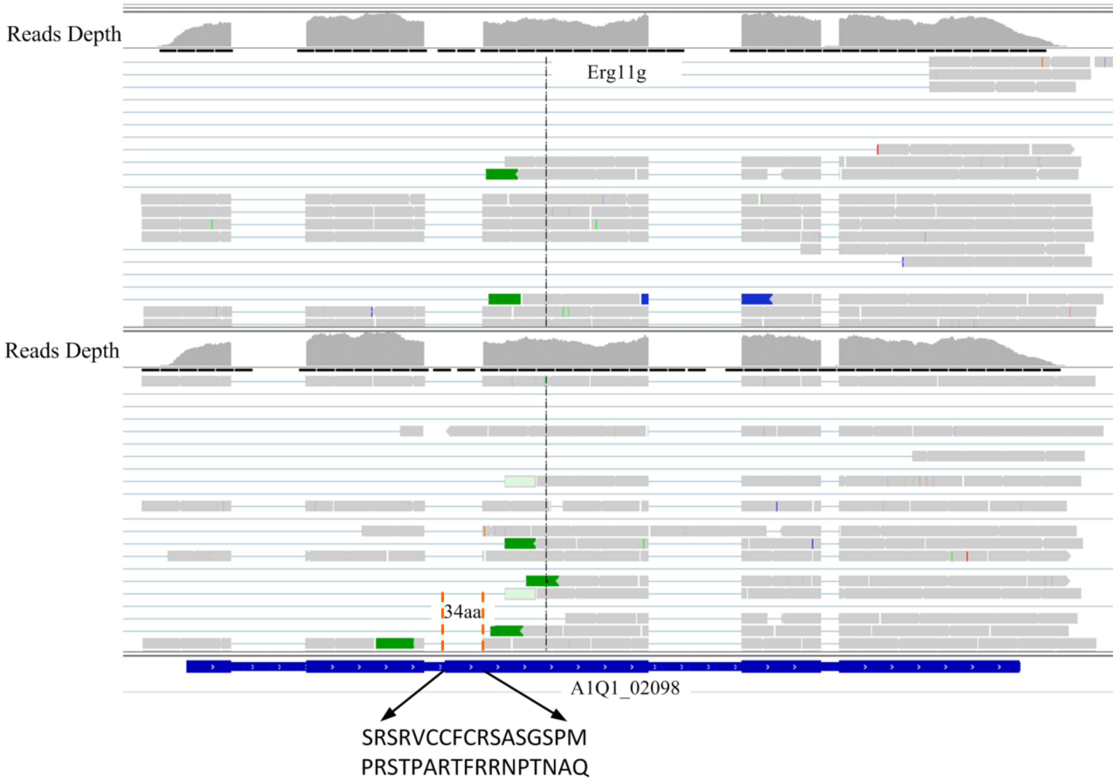

Supplementary Figure S5. Correcting the annotation of A1Q1_02098 exon3. The translated exon3 of A1Q1_02098 is different from its official annotation in the NCBI database. There are 34 amino acids “SRSRVCCFCRSASGSPMPRSTPARTFRRNPTNAQ” (between two red dashed lines) annotated in the NCBI database but no translation signal detected. This transcription signal of its homologous gene Erg11p in *S. cerevisiae* YJM1463 (Identity46%, E-value 3.0e-161) is shown above and also does not have these 34 amino acids. The red dashed line shows the start and end position in exon3. The green and the blue represents the possible deletions (insertions).
